# Comparative genomic mapping of the bovine Fragile Histidine Triad (*FHIT*) tumour suppressor gene: characterization of a 2 Mb BAC contig covering the locus, complete annotation of the gene, analysis of cDNA and of physiological expression profiles

**DOI:** 10.1186/1471-2164-7-123

**Published:** 2006-05-23

**Authors:** Cristina Uboldi, Elena Guidi, Sante Roperto, Valeria Russo, Franco Roperto, Giulia Pia Di Meo, Leopoldo Iannuzzi, Sandrine Floriot, Mekki Boussaha, André Eggen, Luca Ferretti

**Affiliations:** 1 Department of Genetics and Microbiology "A. Buzzati-Traverso", University of Pavia, Italy; 2 Department of Pathology and Animal Health, Faculty of Veterinary Medicine, Naples University "Federico II", Naples, Italy; 3 Laboratory of Animal Cytogenetics and Gene Mapping, National Research Council (CNR), ISPAAM, Naples, Italy; 4 Laboratoire de Génétique biochimique et de Cytogénétique, Département de Génétique Animale, INRA – CRJ, Jouy-en-Josas, France

## Abstract

**Background:**

The Fragile Histidine Triad gene (*FHIT*) is an oncosuppressor implicated in many human cancers, including vesical tumors. *FHIT *is frequently hit by deletions caused by fragility at FRA3B, the most active of human common fragile sites, where *FHIT *lays. Vesical tumors affect also cattle, including animals grazing in the wild on bracken fern; compounds released by the fern are known to induce chromosome fragility and may trigger cancer with the interplay of latent Papilloma virus.

**Results:**

The bovine *FHIT *was characterized by assembling a contig of 78 BACs. Sequence tags were designed on human exons and introns and used directly to select bovine BACs, or compared with sequence data in the bovine genome database or in the trace archive of the bovine genome sequencing project, and adapted before use. *FHIT *is split in ten exons like in man, with exons 5 to 9 coding for a 149 amino acids protein. VISTA global alignments between bovine genomic contigs retrieved from the bovine genome database and the human *FHIT *region were performed. Conservation was extremely high over a 2 Mb region spanning the whole *FHIT *locus, including the size of introns. Thus, the bovine *FHIT *covers about 1.6 Mb compared to 1.5 Mb in man. Expression was analyzed by RT-PCR and Northern blot, and was found to be ubiquitous. Four cDNA isoforms were isolated and sequenced, that originate from an alternative usage of three variants of exon 4, revealing a size very close to the major human *FHIT *cDNAs.

**Conclusion:**

A comparative genomic approach allowed to assemble a contig of 78 BACs and to completely annotate a 1.6 Mb region spanning the bovine *FHIT *gene. The findings confirmed the very high level of conservation between human and bovine genomes and the importance of comparative mapping to speed the annotation process of the recently sequenced bovine genome. The detailed knowledge of the genomic *FHIT *region will allow to study the role of *FHIT *in bovine cancerogenesis, especially of vesical papillomavirus-associated cancers of the urinary bladder, and will be the basis to define the molecular structure of the bovine homologue of FRA3B, the major common fragile site of the human genome.

## Background

*FHIT *is a tumour suppressor gene that is frequently inactivated by deletions in several human tumours, as it overlaps perhaps the most active of common fragile sites FRA3B, at HSA3p14.2 [[1], for a review see [2]]. Infact, *FHIT *is highly responsive to environmental carcinogens, to treatment with aphidicolin – which specifically activates common fragile sites -, and is involved in the development of breast, lung, cervix, stomach, pancreas, head and neck, and kidney tumours, as well as of urothelial tumours, mainly bladder tumours [2]. The FHIT protein is absent or reduced in most cancers, although transcription of the gene is not necessarily altered, as in many malignancies only one allele is lost [3,4]. The human *FHIT *is huge, spanning about 1.5 Mb at the FRA3B locus. However, it codes for a protein of just 147 amino acids, derived by translation of 10 tiny exons of which only exons 5 to 9 contain the ORF; the transcript is very small too, 1.1 kb [1,5].

Fragility at FRA3B and the consequent inactivation of *FHIT *are interesting also because of the presence of an integration site for Human Papilloma Virus mapped to intron 4 [6].

We are investigating *FHIT *in cattle because tumours of the urinary tract in this species are a good model for human carcinogenesis, and because of the suggested involvement of papillomavirus in tumorigenesis [7]. Urinary bladder tumours are a common pathology of 4- to 12-year-old cattle and there is a strong pathogenetic relationship between BPV-2 and some carcinogenic principles of bracken fern [8]. Prolonged ingestion of toxic compounds released by bracken fern, such as ptaquiloside, is responsible for an initial cell transformation of urothelial cells via *RAS *activation; it is believed that papillomavirus infection is associated with tumour progression, whose major clinical symptom is the so called chronic enzootic hematuria (CEH). It is likely that *FHIT *also plays a role in the process [9], and it is an obvious candidate for deletions affecting the locus that should represent the bovine homologue of FRA3B.

In a recent study we isolated an exon tag for bovine *FHIT *which allowed us to map the gene by FISH to BTA22q24 and with radiation hybrids to the telomeric region of BTA22 [10]. Here we used a comparative genomic approach with human *FHIT *to completely annotate the bovine gene. Thus, we describe a 2 Mb 78 BAC contig covering the gene, and present evidence for a very high level of sequence conservation and genomic organization over the whole *FHIT *region between the two species. Finally, we isolated and sequenced four *FHIT *cDNA isoforms, and documented the physiological expression profiles by means of RT-PCR and Northern blot analysis.

This information will be the starting point to study the role of *FHIT *in papillomavirus-associated tumours of bovine urinary bladder, and more in general will help to interpret the implication of *FHIT *in animal carcinogenesis, including the recently hypothesized role in apoptosis of a phosphorylated form of the FHIT protein [11].

## Results and discussion

### Assembly of a BAC contig covering *FHIT*

Short sequence tags derived from the *FHIT *human genomic sequence or taken from the trace archive of the bovine genome sequencing project, were used to isolate by PCR clones from an ordered BAC library [12]. Exon tags were first employed but given the anticipated large size of the genomic region to be covered (1.5 Mb in man), intronic STSs were also used. The STSs that did not amplify comparatively were adapted to the bovine thanks to the homology to non annotated sequence entries available in the bovine genome database (Build 2.1) and bovine genome sequencing trace archive. The whole primer's set is shown in Table 1 [Additional file 1]. As many *FHIT *exons are very short, primers were often designed covering the exon-intron boundaries or spanning the exon from within adjacent introns. In most instances the approach was successful, and unexpectedly also intronic tags positioned very far from exons worked in PCR, supporting once more the high degree of sequence conservation between human and cattle.

By sequential screens of the BAC library with selected primers, a total of 78 BAC clones was retrieved, belonging to three subcontigs. The software Finger Printed Contigs (FPC) was used as explained in Methods to produce the most likely arrangement of the BACs tiling in a single contig. Its structure, shown in Figure [see Additional file 2 ], was subjected to experimental validation using two approaches, 1) amplification of STS tags on the individual BACs, and, 2) alignment of available sequence from the BAC clones (i.e. mapped STS and BAC-ends) to contigs of sequence retrieved from the bovine genome database.

The tiling pattern of BACs in the contig as suggested by FPC was consistent with the PCR mapping data for all the exon and intron tags, except for a group of 13 BACs, labeled with an asterisk in Figure [see Additional file 2]. The inclusion of those BACs in the *FHIT *contig was likely an FPC artefact; indeed, the programme selects the best tiling of a set of clones following a merely statistical evaluation of the similarity in their restriction fingerprints. In our case, however, three lines of evidence allowed to resolve the ambiguity. First, STS-content mapping revealed that none of the 13 BACs contained the expected tags based on the proposed FPC output (i.e. exon 6, 7, 8, intron 8–1 to intron 8–2, see Figure [see Additional file 2]). Second, BACs bI0643H09 and bI0947B04, boxed in Figure [see Additional file 2], could be directly linked in the contig, as they were both positive to the intron 8–1 tag. Third, the sequence of the BAC-ends, available for clones bI0195D05, bI0520A05, and bI0545B04, was BLASTed to the bovine genome database and identified a contig assigned to BTA11 [Genbank:NW_988236], which is extraneous to the *FHIT *bovine locus, previously mapped to BTA22 [10].

In conclusion, the BAC contig covers a region of about 2 Mb spanning the entire *FHIT *locus. The bovine gene is split into 10 exons like the human, separated by introns that appear also similar in size. The only exception to this highly conserved pattern is exon 3 (58 bp in the bovine, 53 bp in the human), which is completely different with respect to sequence and position in the two species. As shown in Figure [see Additional file 2], bovine exon 3 is placed between two intron 2 tags (I2–1, I2–2), while the human's exon 3 position is between I2–6 and I3–1 tags. Another difference in the organization of the bovine gene is the presence of two alternative exons 4 (exon 4A and 4B, Figure [see Additional file 2]) that have no homology in the human *FHIT*. More details on transcripts and alternative splicing are given in a later section.

### VISTA global alignments

To study further the degree of conservation at the *FHIT *locus between the bovine and the human, all the available contigs covering the region were recovered from the bovine genome database (Build 2.1) using representative tags from the BAC clones of the *FHIT *contig previously described. Seven large contigs were retrieved: [Genbank:NW_978846] (314883 bp), containing exon 1; [Genbank:NW_001028446] (64796 bp), containing exon 2 to I2–2 tag; [Genbank:NW_984819] (239821 bp), containing I2–3 to I3–5 tag; [Genbank:NW_930116] (641699 bp), containing I3–6 to I5–2C tag; [Genbank:NW_935020] (49009 bp), containing I5–2 tag; [Genbank:NW_977438] (344934 bp), containing I5–2D to I5–4 tag; [Genbank:NW_968990] (1090058 bp), containing exon 6 to exon 10. The contigs were submitted to the mVISTA server along with the human *FHIT *sequence (Build 36.1) in order to analyse the conservation at the locus. However, as most of the bovine contigs represent largely unfinished raw sequence, e.g. [Genbank:NW_930116] contained two large stretches (67 and 53 kb each) of unassigned base calls, all N runs longer than 1 kb were removed before submitting the contig files to the mVISTA server. In addition, as there is a maximum limit of 2 Mb for the base sequence to be used as reference, the human *FHIT *genomic sequence from HSA3 contig [Genbank:NC_000003.1] was cut between nucleotides 59500000 and 61500000. The *FHIT *gene coordinates are nt 59710076 to 61212164.

**Figure 1:**
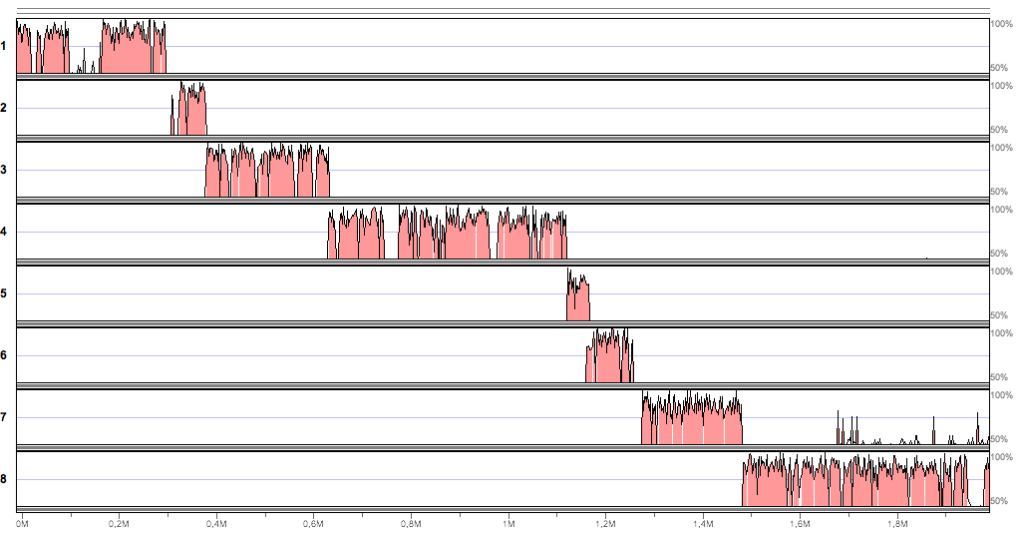
**VISTA Global Alignments of the human and bovine *FHIT *loci**. VISTA plot showing sequence similarity in pairwise sequence alignments between the human sequence at the *FHIT *locus [Genbank:NC_000003.1] nt 59500000 to 61500000; the *FHIT *gene coordinates are nt 59710076 to 61212164] and bovine genomic contigs: 1. [Genbank:NW_978846]; 2. [Genbank:NW_001018446]; 3. [Genbank:NW_984819]; 4. [Genbank:NW_930116_B] (Reverse-complement of the original [Genbank:NW_930116] from nt 84000 to the 3' end); 5. [Genbank:NW_935020]; 6. [Genbank:NW_930116_A] (the initial 84000 nt of the original [Genbank:NW_930116]); 7. [Genbank:NW_977438]; 8. [Genbank:NW_968990]. Peaks are shown relative to their position in the reference human sequence (horizontal axis) and percent identity (50–100%) is shown on the vertical axis. Color code is predominantly pink (non coding sequence) as exons (in blue) are virtually invisible due to their very small size (49 to 317 bp).

The graphical output of the mVISTA server analysis is shown in Figure 1. The bovine contigs are labeled 1 to 8 according to their expected position with respect to the human reference sequence. The conservation of sequence with the human reference sequence was substantially high, in agreement with the data obtained through the STS mapping of the BAC contig described previously. In addition, as the contribution of exons to the whole alignment is negligible (1 kb vs 2 Mb), the VISTA output clearly suggested that non coding sequence was not only conserved (with an average identity well above 70%), but also arranged very similarly between the two species. Thus, the tiling of bovine contigs followed the expected order and did not suggest the existence of rearrangements at the locus between human and bovine, with possibly one exception, represented by contig [Genbank:NW_930116] that required special attention. Indeed, the original contig sequence could be aligned only after, 1) removing two large stretches of 53 and 67 kb of Ns (coordinates 214 to 267 kb, and 453 to 520 kb, respectively) from the sequence and, 2) aligning the 5' 84 kb of the modified contig [Genbank:NW_930116_A in Figure 1] independently from the remaining 84–520 kb portion [Genbank:NW_930116_B in Figure 1], which had also to be reverse-complemented to successfully align. As shown in Figure 1, NW_930116_B was proximal to NW_930116_A, and contig [Genbank:NW_935020] was placed in between them, with a 635 bp overlap at the 5' end with NW_930116_B and a 9 kb overlap at the 3' end with NW_930116_A. This ordering was also in agreement with the tiling of BACs in the contig of Figure [see Additional file 2], and with the PCR mapping of exons and introns tags. An explanation for the apparent rearranged structure of the original contig [Genbank:NW_930116] is that it was formed by merging two sub-contigs (our A and B portions) in the wrong order during the assembly of the whole bovine genome sequence. In this respect, the VISTA alignment clearly suggested that contig [Genbank:NW_935020] might represent a missing portion in the real arrangement of the correct [Genbank:NW_930116] contig. Our interpretation of the arrangement of the seven retrieved genomic contigs is consistent with experimental data, but shall find a final confirmation only when the complete annotation of the bovine genome will be available.

The exon/intron structure of the bovine *FHIT *gene is summarized in Table 2 [see Additional file 3]. The gene coordinates were identified using the seven genomic contigs retrieved from GenBank (Bovine Genome Build 2.1) as reference sequence. The size of the introns could not be defined exactly in all cases, as most contigs still contain large blocks of unfinished sequence; however the predicted size of introns 4 and 5, based on this work, is very close to that of the human gene. This is noticeable, especially for intron 5, that is over 560 Kb, and is known to be the major target of deletions that affect the gene structure in human cancers. It is tempting to speculate that *FHIT *intron 5 might have a similar role also in tumours affecting cattle.

### *FHIT *cDNA isolation and analysis of expression

**Figure 2:**
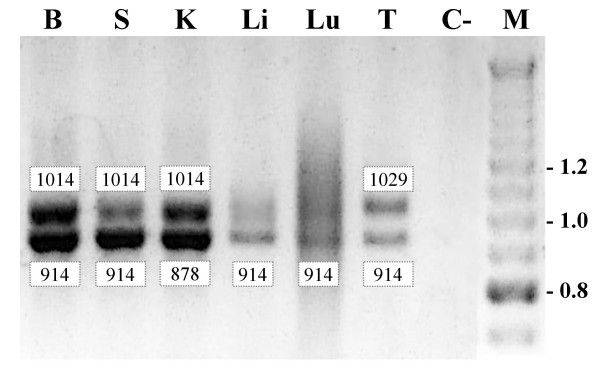
**RT-PCR analysis of *FHIT *expression**. Total RNA extracted from brain (B), spleen (S), kidney (K), liver (Li), lung (Lu) and testis (T) was reverse-transcribed and then amplified with primers designed to reproduce the entire *FHIT *cDNA, i.e. from exon 1 to 10. Numbers close to bands indicate size in bp. C- is a control reaction with no RNA. RT-PCR products were separated on a 1.5% agarose gel. M are DNA size standards (1 Kbp and 100 bp ladder mixed together).

**Figure 3:**
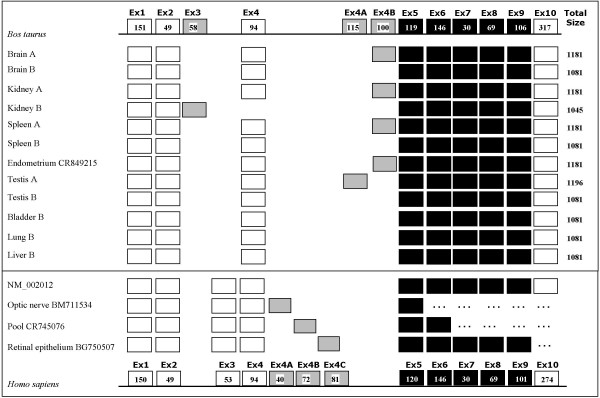
**cDNA isoforms of the *FHIT *gene**. The isoforms of the bovine *FHIT *cDNA identified by RT-PCR analysis in different tissues are listed. Top, schematic of all exons found in the bovine cDNA analysis: non coding (empty boxes), alternatively-used (grey) and coding (black). Size, in bp, is shown for single exons within the box, and at the right for the entire cDNA isoform. Letters (A, B) highlight tissues showing more than one isoform. Endometrium [Genbank:CR849215] is a bovine non annotated cDNA entry found in the NCBI database and homologous to *FHIT*. At the bottom is a schematic for the human *FHIT *gene, with four entries of the NCBI database for the known human cDNA isoforms. [Genbank:BM711354] and [Genbank:CR745076] are 5' RACE products.

In a previous report, we mapped the *FHIT *gene by FISH and radiation hybrids using a bovine EST that matched human exon 9 [10]. Here we designed primers on the human *FHIT *cDNA to cross-amplify the bovine cDNA by means of RT-PCR. Thus, oligonucleotides Exon 1F and Exon 10R (Table 1, [see Additional file 1]), were used to amplify nearly the entire cDNA (expected product size 914 bp), on RNA extracted from brain, spleen, kidney, liver, lung and testis. The results are shown in Figure 2. As expected, *FHIT *is transcribed ubiquitously, and PCR products were obtained in all tissues, although of different sizes. Thus, four bands were visible within the entire set of samples, and were recovered from the gel and sequenced. A band of 914 bp was present in all tissues except kidney, that showed a band of 878 bp. A third product of 1014 bp was found in brain, spleen, and kidney, but not in lung and liver, and a fourth product of 1029 bp was detected in testis. The existence of four different cDNA isoforms is due to an alternative splicing of exons that belong to the 5' untranslated portion of the gene. This was confirmed by sequencing the isoforms of Figure 2 and performing additional RT-PCRs with selected combinations of exon-primers on the same panel of tissues, with the addition of bladder. The results are summarized in Figure 3, with a schematic representation of the exons arrangement and size of the different isoforms. All the isoforms contain the entire coding portion of the *FHIT *mRNA, so they translate into the same polypeptide of 149 amino acids coded by exons 5 to 9. The difference in the RT-PCR products for kidney, spleen and brain (Figure 2) is due to the alternative use of exon 4B (100 bp), yielding a longer cDNA of 1181 bp and a shorter of 1081 bp; the [Genbank:CR849215] endometrium cDNA is a bovine non annotated cDNA entry taken from GenBank that has the same structure of the longer cDNA isoform described in the present study for brain, spleen and kidney (i.e. with exon 4B). Testis shows a different exon 4 variant, 4A (115 bp), thus the cDNA in that tissue is 1196 bp long. A fourth cDNA exists and is the shortest of all: it is the only one with exon 3 (58 bp), but is missing exon 4, so its whole length is 1045 bp. This cDNA is one of the two isoforms detected in the kidney.

For a comparative analysis, human cDNA entries were also retrieved from GenBank. As in the bovine, an alternative use of exons is apparent in the case of the human *FHIT*, as shown in the lower portion of Figure 3. Three isoforms are shown that are characterized by alternative splicing of three exons 4 variants, respectively of 49, 72 and 81 bp, none of which has any homology with the bovine alternative exons 4.

### Northern blot analysis

**Figure 4:**
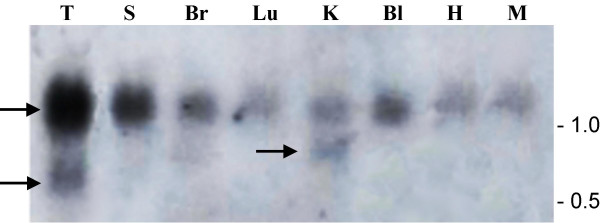
**Mapping of *FHIT *transcripts by Northern blot**. Polyadenylated mRNA extracted from testis (T), spleen (S), brain (Br), lung (Lu), kidney (K), bladder (Bl), heart (H) and muscle (M) was run on a 1.2% agarose formaldehyde gel, blotted onto a nylon filter and hybridized with a labeled *FHIT *probe corresponding to the complete cDNA (exon 1 to 10). The position of RNA size standards is shown at the right; arrows point to the main *FHIT *1.1–1.2 kb transcript and to additional transcripts of 0.6 and 0.8 kb that are visible in testis and kidney, respectively.

The expression of *FHIT *was also monitored by Northern blot analysis with a labeled cDNA probe in the tissues used in RT-PCR (testis, spleen, brain, lung, kidney, bladder), and, additionaly, in heart and muscle (Figure 4). In all samples a major transcript of about 1.1–1.2 kb was visible, a size very close to the known human *FHIT *mRNA, 1095 bp [Genbank:NM_02012]. As already with RT-PCR, *FHIT *was ubiquitously expressed, with apparently higher levels of mRNA in testicular tissue and spleen. However, although equal amounts of polyA mRNA were used, the evidence coming from the Northern blot is only qualitative and cannot be taken as suggestive of variable expression levels between the investigated tissues.

The resolution of the gel used for the Northern blot did not allow to clearly distinguish the mRNA isoforms as in the RT-PCRs of Figure 2. Heart and muscle showed a band of slightly higher molecular weight, but since these tissues were not studied by RT-PCR, it is likely that the major band visible in the Northern blot was a mixture of the cDNA isoforms described before. Only in testis and kidney the probe revealed two additional bands of lower molecular weight, 0.6 and 0.8 kb, respectively. As these mRNA species were not detected with the RT-PCR assay, it is difficult to establish if they were real or rather represented a partial degradation of the *FHIT *mRNA of 1.1–1.2 kb, prior to electrophoresis.

## Conclusion

In this work we used a comparative genomic approach to fully annotate the bovine *FHIT *gene. A set of 78 BACs was assembled in to a genomic contig that covers 2 Mb of DNA. The bovine *FHIT *turned out to have the same arrangement as in man, and an extraordinary conservation of sequence was found throughout all the locus, in such a way that not only the number, but also the size of introns was virtually identical between the two species. The size of the bovine gene could be inferred from the alignment of the BAC contig tags with the 7 genomic contigs recovered from GenBank (Figure [see Additional file 2], and Figure 1), and was around 1.6 Mb from exon 1 to exon 10. Our data allowed to extend the well known finding of a high degree of synteny between the human and bovine genome down to the level of a single gene, including, at least for *FHIT*, the size and nucleotide sequence of introns as large as 560 kb, like intron 5.

Our data contribute to the ongoing bovine genome sequencing project with the annotation of a rather large genomic region. The comparative approach that we used allowed us to highlight inconsistencies and errors in the assembly of bovine genomic contigs spanning the *FHIT *gene region. Studies of this kind will be important to speed up and assist the laborious work of finishing the assembly of the whole bovine genome sequence.

*FHIT *is a very important oncosuppressor gene that lays in the middle of the major common fragile sites of the human genome, FRA3B. The role of the FRA3B/*FHIT *locus in the onset and development of a vast category of important cancers in man has been largely documented. A typical feature of *FHIT *involvement is that rearrangement at the locus gives rise to a complex set of homo- and hemizigous deletions that affect gene function [2]. Among all the tumours showing a loss of FHIT activity, are tumours of the lower urinary tract, especially bladder tumours, one of the most frequent cancers in man. The same situation is likely to exist in cattle tumours, and our complete annotation of the bovine *FHIT *gene will allow to characterize the role of the gene in urothelial tumors, especially of the lower urinary tract. These are a common pathology of 4- to 12-year-old animals grazing in bracken fern (*Pteridium *spp) infested lands [13] and showing Chronic Enzootic Hematuria [14,15].

Using the set of BACs covering the entire bovine *FHIT *region that we describe in the present work, it will be possible to scan tumour samples in search of markers that are lost due to chromosomal breakage, a finding that would confirm the existence of the bovine homologue of the human fragile site FRA3B.

Knowledge of the molecular genomic organization of the *FHIT *locus as gathered from our work will be exploited also to study a peculiar feature of the urinary bladder tumors of cattle. Frequently, such tumors are positive to Bovine Papilloma Virus type-2 (BPV-2), and an intriguing synergy has been suggested between the virus and some clastogenic, mutagenic and carcinogenic compounds released by the bracken fern ingested by animals at pasture. The hypothesis has been made that compounds such as ptaquiloside act synergistically with BPV-2 either in a latent or activated form to produce the neoplastic transformation of cells with a mechanism that remains largely unknown. Thanks to the detailed *FHIT *mapping and annotation achieved in this work, insights into the relation between the presence and activation state of BPV and tumors might come from the study of integration of BPV at the *FHIT *locus. As a matter of fact, we have found a short DNA region in the bovine *FHIT *intron 4, that is homologous to a known integration site of Human Papillomavirus type 16 at *FRA3B*/*FHIT *in man (data not shown) [6].

## Methods

### Design of sequence tags for the selection of BACs covering the bovine *FHIT *region

A bovine BAC containing exon 9 of the gene was already available [10], and was used as a starting point to isolate adjacent clones from a BAC library [12]. In addition, the human *FHIT *genomic sequence [Build 36.1; Genbank:NC_000003.1], nt 59710076 to 61212124 was used as a source of STSs. Primers were designed in exons, as well as in intronic portions of the gene at an average distance of 50 kb in order to conveniently cover the huge target genomic region with PCR-able STSs of a mean size of 250 bp. The whole set of oligonucleotides used is represented in Table 1 [see Additional file 1]. The *FHIT *exons sequences generated have been deposited in GenBank [Genbank:DQ310170-80].

### Assembly of a BAC contig

The screen with the whole set of STSs yielded BAC clones organized in three sub-contigs. The clones were retrieved, the DNA extracted, and *Not*I digests were run on PFGE to estimate the size of inserts. Whithin each sub-contig the end clones were selected in order to look for adjacent overlapping clones. Thus, using the available sequence data for the identified BACs that is posted in GenBank, i.e. the two BAC-ends, primers were designed to amplify the corresponding STSs in the BAC library. Physically assigned STS tags and the output of the Finger Printed Contig (FPC) software tool [16] were combined to progress in the ordering of clones. In this way additional BACs were identified that allowed to link all sub-contigs together, as shown in Figure [see Additional file 2], [17,18].

### Public database searches and identification of bovine contigs containing *FHIT *sequence

The available end-sequence of BACs, and the sequence of all the STS generated and mapped to the bovine *FHIT *contig of BAC clones, were used to search the public database of the bovine genome using BLAST [19]. This yielded 7 contigs: [Genbank:NW_978846 (314883 bp), NW_001018446 (64796 bp), NW_984819 (239821 bp), NW_930116 (641669 bp), NW_935020 (49009 bp), NW_977438 (344934 bp), and NW_968990 (1090058 bp)].

### VISTA global alignments

The mVISTA web server [20,21] was used to align genomic sequences from the bovine genome sequencing project with the human sequence of the *FHIT *locus taken as reference. Thus, the HSA3 sequence [Genbank:NC_000003.1, nt 59500000 to 61500000] spanning the entire *FHIT *gene was aligned with the bovine contigs [Genbank:NW_978846, NW_001018446, NW_984819, NW_930116, NW_935020, NW_977438, and NW_968990]. As many of the bovine sequences are unfinished yet, long runs of unassigned bases were removed prior to analysis; human sequence and its annotation were always used as the reference sequence. Pairwise sequence comparisons were performed with a threshold of 70% identity in a 100 bp window. The VISTA plots showed 50% identity as the minimum value.

### RT-PCR characterization of cDNAs and Northern blot analysis

Total RNA was isolated from testis, spleen, brain, lung, kidney, bladder, heart, and muscle of an healthy cow using Trizol (Invitrogen). For RT-PCR, 3 micrograms RNA was reverse transcribed with SuperScript II (Invitrogen) at 37°C for 2 hours using an oligo-(dT) primer. The subsequent PCR step was performed with oligonucleotides spanning exon 1 to 10 of the gene (Exon 1F and Exon 10R, Table 1 [see Additional file 1]. The primers yield a PCR product of variable size around 1 kb due to the existence of four tissue isoforms (see Results).

For Northern blot analysis, 5 micrograms poly(A) mRNA, purified from    total brain, testis, spleen, lung, kidney, bladder, heart, and    muscle  RNA using a GeneElute mRNA Miniprep Kit (Sigma), was separated on a 1.2% agarose-formaldehyde gel and transferred to HyBond N+ (Amersham). The filter was hybrized with a probe corresponding to the entire *FHIT *cDNA produced as above with primers Exon 1F and Exon 10R (PCR product of 914 bp), and labeled with HexaLabel Plus (MBI Fermentas) in the presence of ^32^P α-dCTP.

The sequence of the four *FHIT *cDNA isoforms has been deposited in GenBank [Genbank:DQ310181-4].

## Authors' contributions

CU and EG developed STS markers, performed the markers assignments to BACs, and finalized the BAC ordering in the final contig. SR, VR and FR organized tissue sample collection and DNA/RNA extraction. GPDM and LI were responsible for the RT-PCR and Northern blot analysis. SF, MB and AE performed the BAC library screening, isolated the BACs and generated with FPC the tiling of BACs in the initial contig. CU and LF performed the sequence analysis and the VISTA global alignments. CU, AE and LF were mainly responsible for conceiving and planning research that was coordinated by LF, who also drafted the first version of the manuscript. All the authors read and approved the final manuscript.

## Supplementary Material

Additional File 1 **Table 1**. A table is presented that describes the sequence and genomic coordinates of all the primers defining the STS tags that were mapped in this work. Click here for file

Additional File 2 **Figure. A BAC contig covering the bovine *FHIT *genomic region. **Top: the BAC clones spanning the bovine *FHIT *locus are aligned with the corresponding region of BTA22 into a 2 Mb contig. The identification of BACs is based on Schibler et al. [16]. The final contig was produced by combining fingerprint data and sequence tags data for BACs of the different subcontigs, with the merging of contigs by means of physically assigned STSs (as shown by vertical lines). Middle: schematic representation of the bovine BTA22 *FHIT *locus region compared to the homologous human HSA3 region shown for reference. The comparison highlights the conservation of individual tags and of the overall locus organization. Exons E1 to E10 are displayed with squares as follows: filled, coding; empty, non coding; grey, alternatively-used. Empty circles are the intronic tags adapted from the human sequence and used in the study. Triangles are three bovine microsatellites. BACs with an asterisk at the right are included by FPC in the contig output but are not linked to the others physically (see text for more details). Bottom: Eight bovine genomic sequence contigs, showing the correspondence with the assembled BAC contig. 1. [Genbank:NW_978846]; 2. [Genbank:NW_001018446]; 3. [Genbank:NW_984819]; 4. and 6. are two independent portions of the original [Genbank:NW_930116] contig separated by contig 5 [Genbank:NW_935020] (for a detailed description of the arrangement of contigs see text and Figure 1); 7. [Genbank:NW_977438]; 8. [Genbank:NW_968990]. The entire contigs are shown, except for [Genbank:NW_978846] and [Genbank:NW_968990] where only the segments that align with the BAC contig are displayed. Click here for file

Additional File 3 **Table 2. Exon/Intron arrangement of the bovine *FHIT *gene**. *FHIT *exons and introns coordinates and size were calculated taking as reference the whole sequence of the bovine genomic contigs retrieved from GenBank (Bovine Genome Build 2.1). *, the contig sequence had to be reverse-complemented to be aligned properly; > the estimated size takes into account the Ns that are present in the unfinished sequence of the corresponding contig, and the Ns that currently separate adjacent contigs; ~, the estimated size may vary according to the number of Ns present in the corresponding contig only; °, The accession numbers for primary sequence data (Ref Seq) within the genomic contigs retrieved from GenBank are shown only for exons, as the number of such sequences for introns would be too high to be included in the table. The complete list of Ref Seq is part of each contig's entry in the GenBank database. The size and sequence of exons was obtained in the present work, see the quoted GenBank accession numbers. Exonic sequence is shown in capital letters. The *FHIT *open reading frame is in bold, with Start and Stop codons underlined. Click here for file
