# Supplementary material for: Comparative genomic mapping of the bovine Fragile Histidine Triad (FHIT) tumour suppressor gene: characterization of a 2 Mb BAC contig covering the locus, complete annotation of the gene, analysis of cDNA and of physiological expression profiles
Source: BMC Genomics. 2006 May 23;7:123. doi: 10.1186/1471-2164-7-123 (PMC1513570; doi:10.1186/1471-2164-7-123)
Supplement: Additional File 1 — Table 1. A table is presented that describes the sequence and genomic coordinates of all the primers defining the STS tags that were mapped in this work. [file 1471-2164-7-123-S1.doc]

**Table 1.** Sequence of the oligonucleotides used to isolate and order the BAC contig covering the bovine FHIT gene.

| **Primer** | **Sequence 5'-3' §** | **Coordinates on contig NC000003** | **Primer** | **Sequence 5'-3' §** | **Coordinates on contig NC000003** |
| --- | --- | --- | --- | --- | --- |
| Exon 1F | TCCCtGCTCTGtTCcGTCCA | 61212164-61212144 | Exon 1R | CTTCCAaGATcTTGgCAGCT | 61212035-61212015 |
| Exon 2F | GAAGCTCAGcAAAGAAGgGA | 61161379-61161359 | Exon 2R | CTTTTGCCTgtGAGGGAGGA | 61161351-61161331 |
| Intr2-1F | GAGGGTGGCCAAATGCAATTG | 61161134-61141113 | Intr2-1R | GGGAACGAAATTAGCTACACC | 61160851-61160830 |
| Exon 3F | ATCtgGcAgGAGAGAAGCAG | 61155568-61155548 | Exon 3R | GCCcTAgATGcAagGCACTT | 61155532-61155512 |
| Intr2-2F | tCACCTCAAGGGAGAGTCAA | 61115477-61115457 | Intr2-2R | TCTGCCTGCAGTAGCCTTCT | 61115223-61115203 |
| Intr2-3F | CCAtgCCAAAacGTTTCATAcg | 61107585-61107563 | Intr2-3R | TATTTGCACCAcGAgTAAGTgG | 61107368-61107346 |
| Intr2-4F | GGCAtGTGAAGATAGGCCcT | 61078949-61078929 | Intr2-4R | TCAGTCACTTGcTCCAAAGGT | 61078815-61078794 |
| Intr2-5F | GATTTCATcCAGTGGCcTGC | 61041604-61041584 | Intr2-5R | ACCCTGACAGCTTTcAAAGtG | 61041514-61041493 |
| Intr2-6F | GCCTGGATGGTACCtAATCAG | 61025809-61025788 | Intr2-6R | ATGCATGTGCCCAAGTCTgAC | 61025603-61025582 |
| Intr3-1F | GAAGTGGTGCTTaGAATGATtG | 60956829-60956807 | Intr3-1R | GTAAgAGGAcTTAttCCCccAA | 60956614-60956592 |
| Intr3-2F | GCATGATTTTGAcCAAGCcTCT | 60934746-60934724 | Intr3-2R | CTCCTTGGGGAATCtAAAGCTT | 60934596-60934574 |
| Intr3-3F | TTAgCCTCTTTCCCTTTGgGT | 60897534-60897512 | Intr3-3R | CATCTTaTCcTAGgcCCAGCA | 60897389-60897367 |
| Intr3-4F | AATaCTGGAAtGATGGGcTCCA | 60877448-60877426 | Intr3-4R | TCATTACCtACTGCAcCACTCT | 60877279-60877257 |
| Intr3-5F | GACTgGGGTTTCACAaGACTC | 60829011-60828990 | Intr3-5R | AGCAGCAgGAGAATGAGCTAC | 60828840-60828819 |
| Intr3-6F | CgACAAGGATGccTTCTAATTG | 60801918-60801896 | Intr3-6R | CCtgAAAcCCAtTTCTTCATCC | 60801698-60801677 |
| Exon 4F | GCTCAGTATTCATGcTGAAt | 60782745-60782725 | Exon 4R | AACCTACCTgcCtGAAGACT | 60782673-60782653 |
| Exon 4AF | ATCTTGATCCGTAGGGCCAC | NW-436453 * | Exon 4AR | CAGATCTTTGGAGAACGTGGC | NW-436453 * |
| Exon 4BF | GGACTCTCTGGGTTTCATGC | NW-274627 * | Exon 4BR | GCAGGAAAAACAGGAGCAGG | NW-274627 * |
| Exon 5F | ATGTCGTTCAGATTTGGCCAAC | 60497752-60497733 | Exon 5R | GTCCaGGTACCACAGGcTT | 60497652-60497633 |
| Intr5-1F | TGACCTGCTTcCCATGCAAG | 60490425-60490405 | Intr5-1R | CTgCCTACTGATGTTCtAAC | 60490223-60490203 |
| Intr5-2F | CTCTTTTcaGgTGGAAGGAT | 60339966-60339946 | Intr5-2R | CCTGgCTgAAACTGGTcTTG | 60339741-60339721 |
| Intr5-2AF | GTGGTTGGACTGCTAATGAGA | 60308002-60307981 | Intr5-2AR | ATTCCCAATCcaCCTACCAAC | 60307847-60307826 |
| Intr5-2BF | CATCTGGCAAAGAGGCAGAT | 60277013-60276993 | Intr5-2BR | ACCTcTtCTGACTgACcgCA | 60276707-60276687 |
| Intr5-2CF | TATCTGCcGTAAccGCTCCTT | 60250525-60250504 | Intr5-2CR | CCTGAAGCcGACTTGAAAGTC | 60250365-60250344 |
| Intr5-2DF | CTCCCATTAGGAAAACCCCAG | 60215528-60215507 | Intr5-2DR | TCgAgAGGAcTAAcGTGGTAT | 60215319-60215298 |
| Intr5-3F | AaGGAGTAGAGGATAgTGCAg | 60177611-60177590 | Intr5-3R | CTaCTAATCCTATgGCTtGTA | 60177391-60177370 |
| Intr5-4F | CGTtTGAAATGTgCAtGGGAG | 60056173-60056152 | Intr5-4R | GGAGGCAAAACTTCTatagCT | 60055974-60055953 |
| Exon 6F | ATGTCCTcGTGTGtCCcCT | 59974918-59974899 | Exon 6R | CCTGCATGGAAAAcGTGAG | 59974792-59974773 |
| Exon 7F | AGCAGGAGATACCATCTATCTg | 59972166-59972144 | Exon 7R | GTCTCTCTGACCTCGAAGATAA | 59972159-59972137 |
| Exon 8F | GaCTTCAcGAGAGCATCgCT | 59883180-59883160 | Exon 8R | CAgTAAGATCAGAGAgAGCA | 59883132-59883112 |
| Intr8-1F | TGGGTGACATTGAATGGTTG | 59878160-59878140 | Intr8-1R | TgCAGCtCTCAGGGCCAAAT | 59877890-59877870 |
| Intr8-AF | GGTATCTCTCCTTCAGGCTAc | 59848742-59848721 | Intr8-AR | AAGcGtCTCCgAACTCAGCAA | 59848571-59848550 |
| Intr8-BF | TCcGACATAAAGGCTTCCCAT | 59810889-59810869 | Intr8-BR | ATCCTTCTCCCCATGAAGcAa | 59810724-59810704 |
| Intr8-CF | TAAGACCTTCcCCtTCcAGG | 59786206-59786186 | Intr8-CR | gACcGCCTcAAAGaTAAgCC | 59785959-59785939 |
| Intr8-2F | ATgGCCAgAaTTGCcAGAGG | 59826268-59826247 | Intr8-2R | GTTCATAgTTCTTCTCtgGA | 59826108-59826087 |
| Intr8-3F | AAGAGTCtcGTcGAGTTCCAG | 59761107-59761086 | Intr8-3R | TGCCgAGAAGTGTgACTaTGC | 59760894-59760873 |
| Intr8-4F | CgATGGAGCATTcATCATcTG | 59721203-59721182 | Intr8-4R | GGCAATCaGCTTGACAAcGTG | 59720905-59720884 |
| Exon 9F | TCAGAGGAGGAAATGGCAGC | 59713087-59713067 | Exon 9R | GTCACTGAAAGTAGACCCTC | 59713007-59712987 |
| Exon 10F | GAacAAATCCCCAGGCATAAG | 59710340-59710319 | Exon 10R | TTGAAACATAACCCCGTTGGC | ** |

§ Primers were selected based on their position in the human FHIT contig (NC-000003) and confronted with the trace archive database of sequencing runs of the bovine genome project to verify their homology. In case of identity the oligonucleotides were used as such; otherwise the sequence identified in the bovine trace archive was used. To reflect changes in the sequence of primers between the two species the affected nucleotides are shown in lower case.

* In this case a bovine sequence entry from GenBank was used directly to design the primers

** Exon 10 in the bovine is longer than in man, so here the primers were designed directly on a sequence from the bovine trace archive
