# Supplementary material for: Comparative genomic mapping of the bovine Fragile Histidine Triad (FHIT) tumour suppressor gene: characterization of a 2 Mb BAC contig covering the locus, complete annotation of the gene, analysis of cDNA and of physiological expression profiles
Source: BMC Genomics. 2006 May 23;7:123. doi: 10.1186/1471-2164-7-123 (PMC1513570; doi:10.1186/1471-2164-7-123)
Supplement: Additional File 2 — Figure. A BAC contig covering the bovine FHIT genomic region. Top: the BAC clones spanning the bovine FHIT locus are aligned with the corresponding region of BTA22 into a 2 Mb contig. The identification of BACs is based on Schibler et al. [16]. The final contig was produced by combining fingerprint data and sequence tags data for BACs of the different subcontigs, with the merging of contigs by means of physically assigned STSs (as shown by vertical lines). Middle: schematic representation of the bovine BTA22 FHIT locus region compared to the homologous human HSA3 region shown for reference. The comparison highlights the conservation of individual tags and of the overall locus organization. Exons E1 to E10 are displayed with squares as follows: filled, coding; empty, non coding; grey, alternatively-used. Empty circles are the intronic tags adapted from the human sequence and used in the study. Triangles are three bovine microsatellites. BACs with an asterisk at the right are included by FPC in the contig output but are not linked to the others physically (see text for more details). Bottom: Eight bovine genomic sequence contigs, showing the correspondence with the assembled BAC contig. 1. [Genbank:NW_978846]; 2. [Genbank:NW_001018446]; 3. [Genbank:NW_984819]; 4. and 6. are two independent portions of the original [Genbank:NW_930116] contig separated by contig 5 [Genbank:NW_935020] (for a detailed description of the arrangement of contigs see text and Figure 1); 7. [Genbank:NW_977438]; 8. [Genbank:NW_968990]. The entire contigs are shown, except for [Genbank:NW_978846] and [Genbank:NW_968990] where only the segments that align with the BAC contig are displayed. [file 1471-2164-7-123-S2.ppt]

## Slide 1
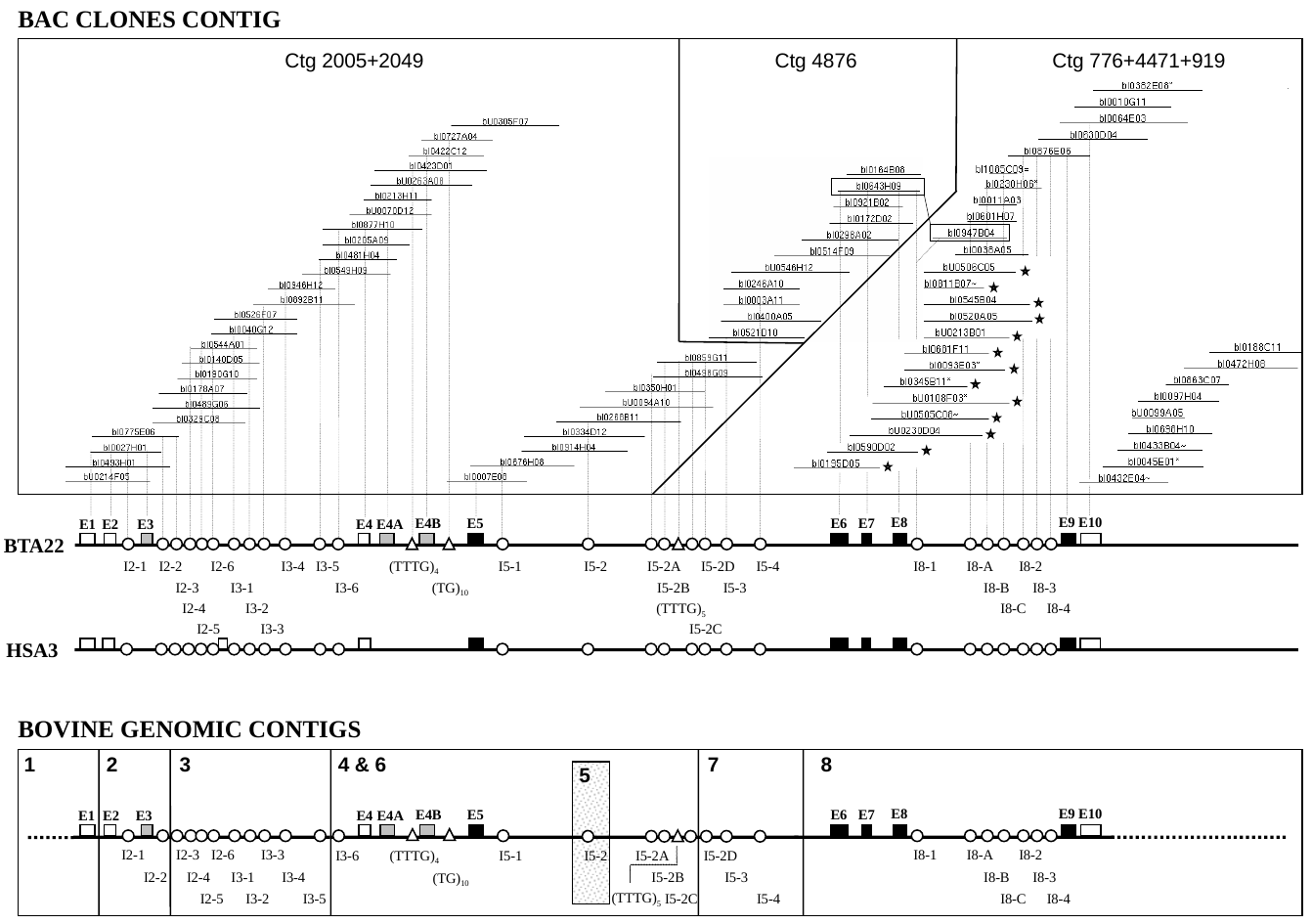

BAC CLONES CONTIG
Ctg 2005+2049
Ctg 4876
Ctg 776+4471+919
E9
E10
E8
E4B
E5
E6
E7
E1
E2
E3
E4
E4A
BTA22
I2-1
I2-2
I2-6
I3-4
I3-5
(TTTG)4
I5-1
I5-2
I5-2A
I5-2D
I5-4
I8-1
I8-A
I8-2
I2-3
I3-1
I3-6
(TG)10
I5-2B
I5-3
I8-B
I8-3
I2-4
I3-2
(TTTG)5
I8-C
I8-4
I2-5
I3-3
I5-2C
HSA3
BOVINE GENOMIC CONTIGS
1
2
3
4 & 6
7
8
5
E9
E10
E8
E6
E7
E4B
E5
E4
E4A
E1
E2
E3
I8-1
I8-A
I8-2
I2-1
I2-3
I2-6
I3-3
I3-6
(TTTG)4
I5-1
I5-2
I5-2A
I5-2D
I8-B
I8-3
I2-2
I2-4
I3-1
I3-4
I5-2B
I5-3
(TG)10
(TTTG)5
I8-C
I8-4
I2-5
I3-2
I3-5
I5-2C
I5-4
